# Whole-body MRI quantitative biomarkers are associated significantly with treatment response in patients with newly diagnosed symptomatic multiple myeloma following bortezomib induction

**DOI:** 10.1007/s00330-017-4907-8

**Published:** 2017-06-27

**Authors:** Arash Latifoltojar, Margaret Hall-Craggs, Alan Bainbridge, Neil Rabin, Rakesh Popat, Ali Rismani, Shirley D’Sa, Nikolaos Dikaios, Magdalena Sokolska, Michela Antonelli, Sebastien Ourselin, Kwee Yong, Stuart A. Taylor, Steve Halligan, Shonit Punwani

**Affiliations:** 1 Centre for Medical Imaging, University College London, 3rd Floor, Wolfson House, 4 Stephenson Way, London, UK NW1 2HE; 2 Department of Radiology, University College London Hospital, London, UK; 3 Department of Medical Physics and Bioengineering, University College London Hospital, London, UK; 4 Department of Haematology, University College London Hospital, London, UK; 5 Translational Imaging Group, Centre for Medical Imaging Computing, University College London, London, UK

**Keywords:** MRI, Whole body, Multiple myeloma, Bortezomib, Response monitoring

## Abstract

**Objectives:**

To evaluate whole-body MRI (WB-MRI) parameters significantly associated with treatment response in multiple myeloma (MM).

**Methods:**

Twenty-one MM patients underwent WB-MRI at diagnosis and after two cycles of chemotherapy. Scans acquired at 3.0 T included T2, diffusion-weighted-imaging (DWI) and mDixon pre- and post-contrast. Twenty focal lesions (FLs) matched on DWI and post-contrast mDixon were selected for each time point. Estimated tumour volume (eTV), apparent diffusion coefficient (ADC), enhancement ratio (ER) and signal fat fraction (sFF) were derived. Clinical treatment response to chemotherapy was assessed using conventional criteria. Significance of temporal parameter change was assessed by the paired *t* test and receiver operating characteristics/area under the curve (AUC) analysis was performed. Parameter repeatability was assessed by interclass correlation (ICC) and Bland–Altman analysis of 10 healthy volunteers scanned at two time points.

**Results:**

Fifteen of 21 patients responded to treatment. Of 254 FLs analysed, sFF (*p* < 0.0001) and ADC (*p* = 0.001) significantly increased in responders but not non-responders. eTV significantly decreased in 19/21 cases. Focal lesion sFF was the best discriminator of treatment response (AUC 1.0). Bone sFF repeatability was excellent (ICC 0.98) and better than bone ADC (ICC 0.47).

**Conclusion:**

WB-MRI derived focal lesion sFF shows promise as an imaging biomarker of treatment response in newly diagnosed MM.

***Key Points*:**

• *Bone signal fat fraction using mDixon is a robust quantifiable parameter*

• *Fat fraction and ADC significantly increase in myeloma lesions responding to treatment*

• *Bone lesion fat fraction is the best discriminator of myeloma treatment response*

## Introduction

Multiple myeloma (MM) is a heterogeneous disease where tumour deposits occur commonly within the bone marrow (BM) of the axial and appendicular skeletons.

Over the past decade whole-body MRI (WB-MRI) has developed as a new modality for cancer detection. In myeloma WB-MRI is superior to skeletal survey, computed tomography, positron emission tomography and whole spine MRI for pretreatment assessment [1–4]. The International Myeloma Working Group (IMWG) consensus guidelines and the National Institute of Care and Health Excellence (NICE) have recommended WB-MRI as the imaging modality of choice for pretreatment assessment of plasma cell disorders [5, 6].

Despite availability of imaging methods for staging disease, treatment response in myeloma is assessed using global indirect measures including serum and urine M-protein levels, serum free light chain ratio and percentage of clonal plasma cells in bone marrow biopsy [7].

Anatomical MRI sequences depict the pattern of BM involvement and characterise the number/size of focal lesions, providing diagnostic/prognostic information in MM [8, 9]. Change in disease pattern/visualization following treatment on anatomical MRI has some value in monitoring response, but remains subjective and inconsistent [9, 10]. Addition of functional imaging biomarkers [11, 12] may provide quantitative monitoring of treatment response and potentially indicate outcome at early stages of treatment [13]. Indeed, quantitative measurements from dynamic contrast-enhanced (DCE) and diffusion-weighted (DWI) MR imaging show promising results for disease characterization and response monitoring in MM [14–17].

However, there is as yet no agreed methodology for imaging-based quantitative evaluation of treatment response in MM; moreover, repeatability of currently proposed biomarkers remains challenging. There remains a need to define a reliable, sensitive and specific biomarker that can be used for whole-body imaging, providing an assessment of heterogeneity of treatment response across skeletal lesions.

The purpose of this study was to evaluate WB-MRI quantitative biomarkers of treatment response for patients with symptomatic MM.

## Methods

A prospective single-arm observational study was conducted. Institutional ethics permission was obtained (REC Number: 12/LO/0428). Participants were recruited from a single tertiary centre and gave written informed consent.

### Patient cohort

Thirty patients were prospectively identified from multidisciplinary team meetings (consisting of a radiologist, pathologist and haematologist) between June 2012 and September 2014 inclusive (M/F 13:17, median age 55, range 36–82 years). Inclusion criteria were age at least 18 years; clinical suspicion of symptomatic MM; no previous malignancy, chemotherapy or radiotherapy; eGFR greater than 50 mL/min/1.73 m^2^; no contraindication to MRI.

Prior to chemotherapy, all patients (*n* = 30) underwent baseline WB-MRI. Symptomatic MM patients (*n* = 26) were treated with 4–8 cycles of bortezomib-based chemotherapy and repeat WB-MRI performed at the end of the second cycle.

Treatment response was evaluated by the clinical team, blinded to imaging results, on the basis of International Myeloma Working Group consensus after bortezomib induction [7] and patients classified into ‘responder’ and ‘non-responder’ groups based on achieving a minimum of partial response.

All non-responders were escalated to salvage chemotherapy treatment with bortezomib, thalidomide and dexamethasone (VTD) whilst responding patients went on to have immediate (*n* = 7) or deferred (*n* = 8 as part of the PADIMAC trial: EudraCT 2010-021598-35) autologous stem cell transplant.

**Fig. 1:**
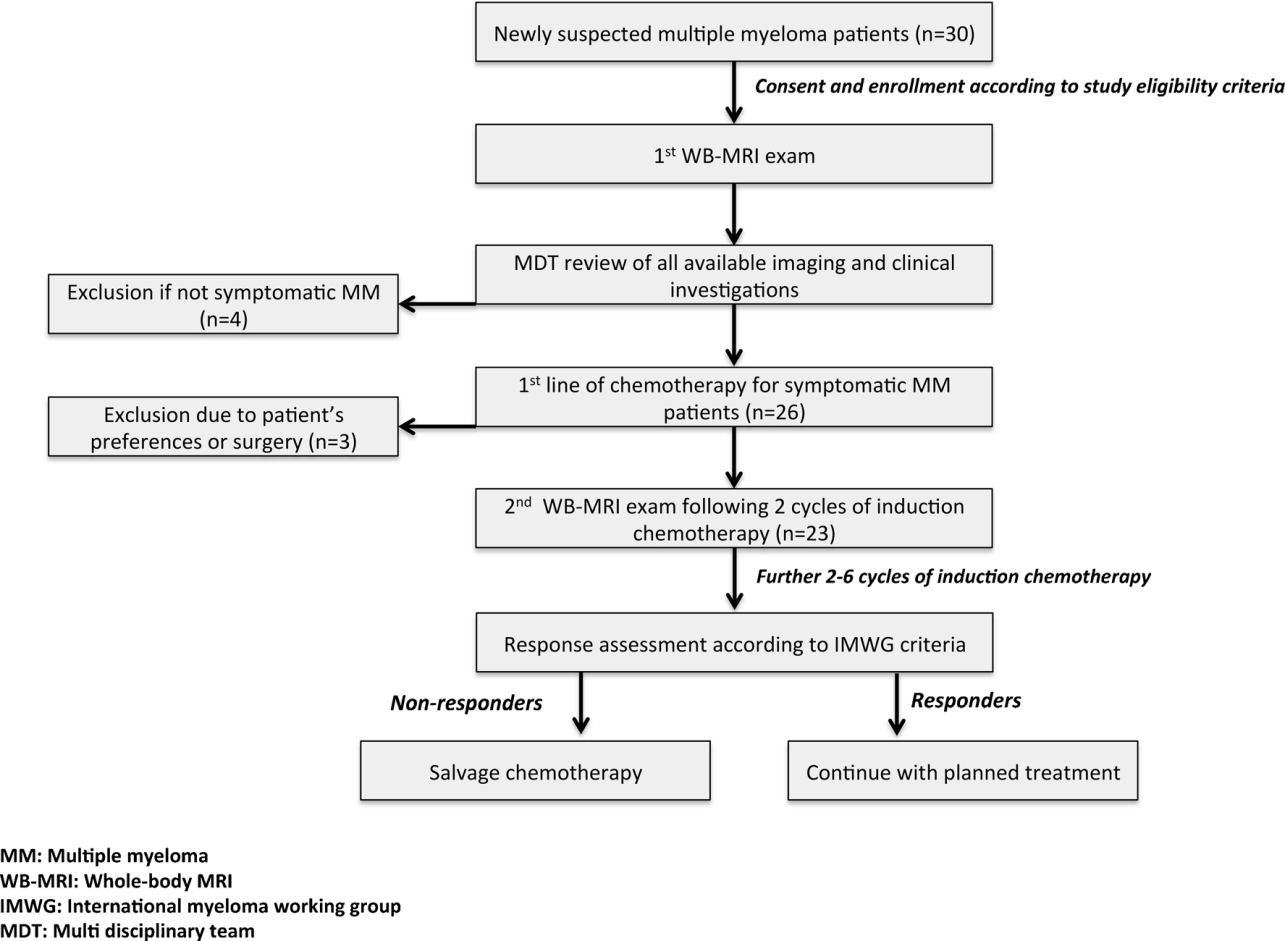
Patient selection and recruitment flowchart

Figure 1 presents the patient selection flowchart.

### Volunteer cohort

Repeatability of WB-MRI quantitative metrics was assessed, by repeating WB-MRI at a median of 4 weeks (range 1–11 weeks) between 1st and 2nd scans, in 10 healthy volunteers (M/F 6:4, median age 33 years, range 24–39 years) recruited from our institutional employees between January 2013 and October 2014.

### WB-MRI technique

Imaging was performed using a single 3.0-T wide-bore MR scanner (Ingenia; Phillips Healthcare, Best, Netherlands). Full body coverage (vertex to feet) was obtained through a multistation acquisition of contiguous body regions with the manufacturers’ head coil, two anterior surface coils and table-embedded posterior coils. Whole-body coronal pre-contrast mDixon imaging was complemented by axial T2-weighted turbo spin echo (TSE), axial DWI (with four *b* values, *b*_0,100,300 and 1000_) and finally, coronal contrast-enhanced (CE) mDixon imaging. CE MRI was performed following 20 ml of intravenous (IV) gadoterate meglumine (Dotarem, Guebert, France) injection. The average total scan time was 67 min. For mDixon imaging a 3D gradient echo sequence with two echoes acquired at first out-of-phase and first in-phase time of echoes was used, to maximise signal-to-noise ratio and minimise effect of T2* decay. Post-processing of attained out-of-phase and in-phase images using scanner software created fat-only and water-only images. The same procedure was repeated following contrast agent injection (CE mDixon).

CE mDixon was not performed in the volunteer cohort as the majority of participants did not consent to IV contrast injection.

Full scanning parameters are summarised in Table 1.

**Table 1:** MRI sequence parameters

	T2-TSE	mDixon (pre and post-contrast*)	DWI (b0, 100, 300, 1000)
Imaging plane	Transverse	Coronal	Transverse
TE (ms)	80	1.02/1.8	71
TR (ms)	1228	3.0	6371
FOV (mm × mm)	500 × 300	502 × 300	500 × 306
Voxel size (mm × mm)	1 × 1	2.1 × 2.1	4 × 4.2
Number of slices	40	120	40
Slice thickness (mm)	5	5	5
Acquisition matrix	500 × 286	144 × 238	124 × 72
ETL	91	2	39
Acceleration factor (SENSE)	2	2	2.5
Pixel bandwidth (Hz)	537	1992	3369
Acquisition time per station (s)	47	17	152
Number of stations	9	5 (6)	9
Total FH coverage (mm × mm)	1777.95 (10% overlap)	1736 (10% overlap)	1777.95 (10% overlap)

*T2*-*TSE* T2-weighted turbo spin echo, *mDixon* modified Dixon, *DWI* diffusion weighted imaging, *TE* time of echo, *TR* time of repetition, *ETL* echo train length, *SENSE* sensitivity encoding

*Contrast agent 20 ml intravenous gadoterate meglumine (Dotarem, Guerbet, France)

### WB-MRI quantitative analysis

#### Patient cohort

Two radiologists (MHC and SP with over 25 and 10 years of experience in MR imaging, respectively) reviewed images in consensus and blinded to other investigations. The pattern of BM involvement was noted for each patient using a combination of all available sequences as previously reported [18].

FLs in 10 contiguous anatomical locations (skull, cervical spine, shoulder girdle, humerus, chest wall, thoracic spine, lumbar spine, pelvis, femur and lower leg) were localized on WB-DWI and CE water-only mDixon MRI.

FLs were identified as focal areas of restricted diffusion returning high signal intensity on DWI *b*_1000_ images and increased contrast uptake on CE water-only mDixon images compared to surrounding marrow. A 5-point scale was used to score confidence of lesion presence (0, non-diagnostic; 1, unlikely; 2, indeterminate; 3, likely; and 4, highly likely disease).

As a result of the heterogeneous nature of MM and its tendency to involve the entire skeleton, and in order to provide a practical and comparable global measure of disease across bony sites, a maximum of 20 FLs per patient were selected on the basis of the following applied rules:

I. Included only lesions of at least 5 mm, scored 3/4 and visible on anatomically matched *b*_1000_ DWI and CE water-only mDixon
II. Included all lesions from anatomical locations with less than three lesions
III. Included largest lesions from other sites, to a make up for a maximum of 20 FLs per patient based on current Durie–Salmon PLUS staging criteria [19]

#### Biomarker quantitation

Analysis was performed using Osirix (Version 4.0, Apple, California, USA).

For quantitative analysis, a single slice containing the FL’s largest dimension was indicated by two reporting radiologists (MHC and SP) in consensus who derived the quantitative parameters as below.

*Estimated tumour volume* (*eTV*) (*cm* ^*3*^): Individual lesion volume was calculated from three-axis measurements on CE water-only mDixon (*X*/*Y*) and *b*_1000_ DWI (*Z*) images; eTV was calculated as (*X* × *Y* × *Z*/2).

*Enhancement ratio* (*ER*) (%): Average signal intensity (SI) was measured for lesions on pre- and post-CE water-only mDixon images and ER calculated as [(SI _post-contrast-W_ − SI _pre-contrast-W_)/SI _pre-contrast-W_] × 100.

*Apparent diffusion coefficient* (*ADC*) (×10^−3^ mm^2^/s): Average signal intensity of lesions was derived for each *b* value. ADC was derived using a mono-exponential curve fit implemented in MATLAB 2011a (MathWorks, Massachusetts, USA) [20].

*Signal fat fraction* (*sFF*) (*a.u*.): sFF was derived from average signal intensity of on pre-contrast water and fat-only mDixon images. sFF = SI _pre-contrast-F_/(SI _pre-contrast-F_ + SI _pre-contrast-W_) [21].

Finally, and as an internal control within the patient cohort, the sFF of the greater trochanter (involved uncommonly by myeloma) was calculated at each time point using a 3 cm^2^ region of interest (ROI).

#### Volunteer cohort

sFF and ADC repeatability was assessed on matched coronal mDixon and ADC maps by a single observer. For each volunteer, seven single slice skeletal ROIs (T10 and L4 vertebral bodies, sacroiliac joint and sacral ala, iliac crest, femoral head and neck, mid-shaft femur and distal femur), a 2 cm^3^ circular ROI of the spleen on ADC maps and a 3 cm^3^ circular ROI of subcutaneous adipose tissue at the level of right femoral greater trochanter on mDixon MRI images were measured at two time points using Osirix (Version 4.0, Apple, California, USA).

### Statistical analysis

#### Grouped analysis

Distribution normality was assessed by the Kolmogorov–Smirnov test. Mean lesion values of MRI biomarkers (eTV, ER, ADC and sFF) were derived for both groups, for baseline and post-2nd cycle scans. Changes of MRI biomarkers between baseline and post-2nd cycle scans were assessed for each group by the two-tailed paired *t* test.

The two-tailed Mann–Whitney test was used to compare lesion count between responder and non-responder groups. Statistically significant differences were defined as *p* < 0.05.

#### Per patient analysis

Median lesion values of MRI biomarkers across all selected FLs were derived for each individual patient for baseline and early post-treatment studies. Changes of MRI biomarkers between the two time points were assessed for each patient by the two-tailed Wilcoxon matched-pairs signed-rank test. Statistically significant differences were defined as *p* < 0.05.

Receiver operating characteristics (ROC) curves, for prediction of non-responding patients, were derived for each MRI biomarker at each time point, and for percentage change in each MRI biomarker following treatment ([early post-treatment − baseline] × 100/baseline). The area under the curve (AUC) was quantified to assess performance for discriminating non-responding patients across all possible diagnostic thresholds.

#### Biomarker repeatability (volunteer cohort)

Repeatability of sFF and ADC of bone, ADC of spleen and sFF of subcutaneous adipose tissue was assessed by one-way random single measure interclass correlation coefficient (ICC) statistics and Bland–Altman plots.

Statistical analysis was performed using Prism software (Prism Version 6.0, GraphPad, California, USA) by the study clinical research fellow (AL).

## Results

### Patient cohort

Four patients (4/30) did not have symptomatic MM and were excluded (one monoclonal gammopathy of uncertain significance, one asymptomatic multiple myeloma, two solitary plasmacytoma).

Three patients (3/26) did not have the second scan because of interval surgery or patient preference. Two further patients with post-2nd cycle scans were excluded from quantitative analysis as one underwent radiotherapy and a second had no FL. MRI biomarkers were evaluated in the remaining 21 patients.

Demographics, blood tests, staging and treatment regimens for 21 patients are summarised in Table 2.

**Table 2:** Patient cohort: demographics, routine blood tests, staging and chemotherapy regimen

Patient characteristic (*N* = 21)	Number or median (range)
Age	55 (36–69)
Sex	Male/female: 13/8
Chain isotype	
IgG	14
IgA	4
Light chain	3
ISS stage	
I	6
II	12
III	3
DS-PLUS stage	
I	1
II	2
III	18
Induction regimen	
PAD	16
CVD	3
VTD	2
Bone marrow percentage plasma cells	65 (15–90)
Beta-2 microglobulin (mg/L)	3.8 (1.3–10.3)
Albumin (g/L)	40 (32–53)
Creatinine (μmol/L)	79 (58–105)

*ISS* international staging system, *DS*-*PLUS* Durie–Salmon PLUS, *PAD* bortezomib, doxorubicin, dexamethasone, *CVD* cyclophosphamide, bortezomib, dexamethasone, *VTD* bortezomib, thalidomide, dexamethasone

Fourteen (14/21) patients had focal only and seven (7/21) patients diffuse and focal pattern of involvement. Fifteen of 21 patients achieved a minimum of partial response (PR) after induction chemotherapy [3/15 with PR, 3/15 with complete response (CR) and 9/15 with very good partial response (VGPR)] and were collectively classified as the responder group. Six of 21 patients achieved less than PR (<PR) to induction chemotherapy [4/6 with minimal response (MR), 1/6 with stable disease (SD) and 1/6 with progressive disease (Prog)] and were collectively classified as non-responders.

### Grouped analysis

The median number of lesions evaluated per patient was 14 (range 1–18). A total of 186 (median 14, range 1–18) and 68 (median 12, range 7–15) FLs were evaluated in responder and non-responder groups respectively (*p* = 0.33).

Mean [standard deviation (SD)] eTV, ADC, sFF and ER at baseline and post-2nd cycle for responders and non-responders is shown in Table 3.

**Table 3:** Patient cohort: mean and standard deviation of imaging biomarker distribution at baseline and post-2nd cycle in responder and non-responder groups

	Scan	Mean eTV (SD)	Mean ER (SD)	Mean ADC (SD)	Mean sFF (SD)
Responders (*n* = 15)	Baseline	0.41 (0.36)	142 (101.40)	0.89 (0.32)	0.28 (0.11)
	Post-2nd cycle	0.28 (0.30) ^a^	142 (67.30)	1.34 (0.49) ^a^	0.52 (0.16) ^a^
Non-responders (*n* = 6)	Baseline	0.52 (0.53)	107.5 (41.77)	0.59 (0.15)	0.40 (0.14)
	Post-2nd cycle	0.29 (0.28)	131.5 (72.46)	0.78 (0.41)	0.43 (0.17)

*eTV* estimated total tumour volume, *ER* enhancement ratio, *ADC* apparent diffusion coefficient, *sFF* signal fat fraction, *SD* standard deviation

^a^Significant change (*p* < 0.05) compared with baseline scan

**Fig. 2:**
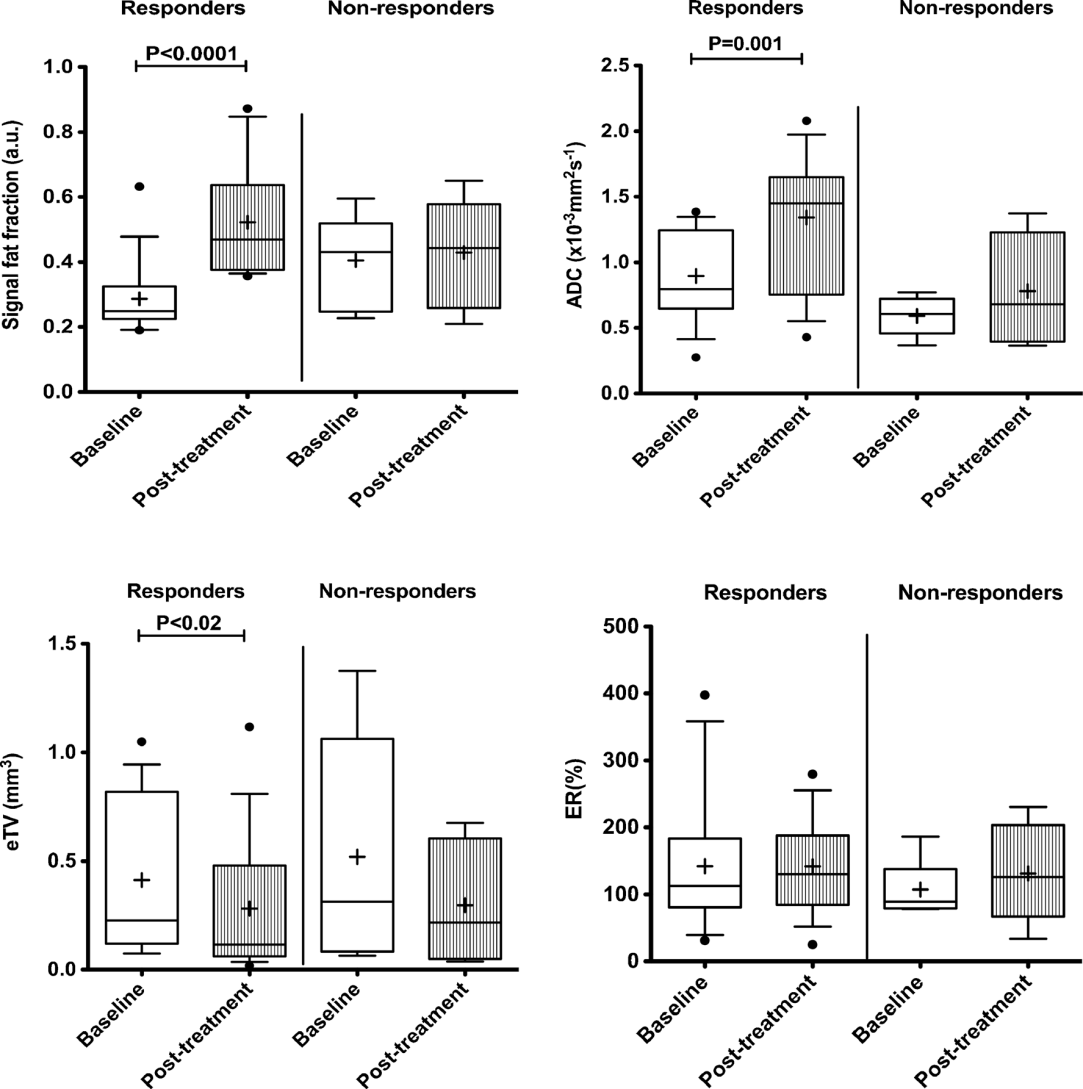
Box and whisker plots of temporal changes of **a** signal fat fraction (sFF), **b** apparent diffusion coefficient (ADC), **c** estimated tumour volume (eTV) and **d** enhancement ratio (ER) in responder and non-responder groups. The *boundaries of the box* show 25th and 75th percentiles, and the *line within the box* is the median. *Whiskers* show 10th and 90th percentiles. Means (+) and outliers (•) are shown. Each *point* represents a patient

Following treatment, there was a significant reduction in eTV in responding groups (*p* < 0.02) whilst no significant changes were observed in non-responder group (*p* = 0.09). sFF increased significantly for the responder group (*p* < 0.0001), but not for the non-responder group (*p* = 0.21). Similarly, ADC significantly increased in responders (*p* = 0.001) but not in non-responders (*p* = 0.15). No significant change was observed for ER for either group (*p* = 0.99 and 0.48 for responders and non-responders, respectively). Group-based analysis for temporal changes of each biomarker is presented in Fig. 2.

### Per patient analysis

**Fig. 3:**
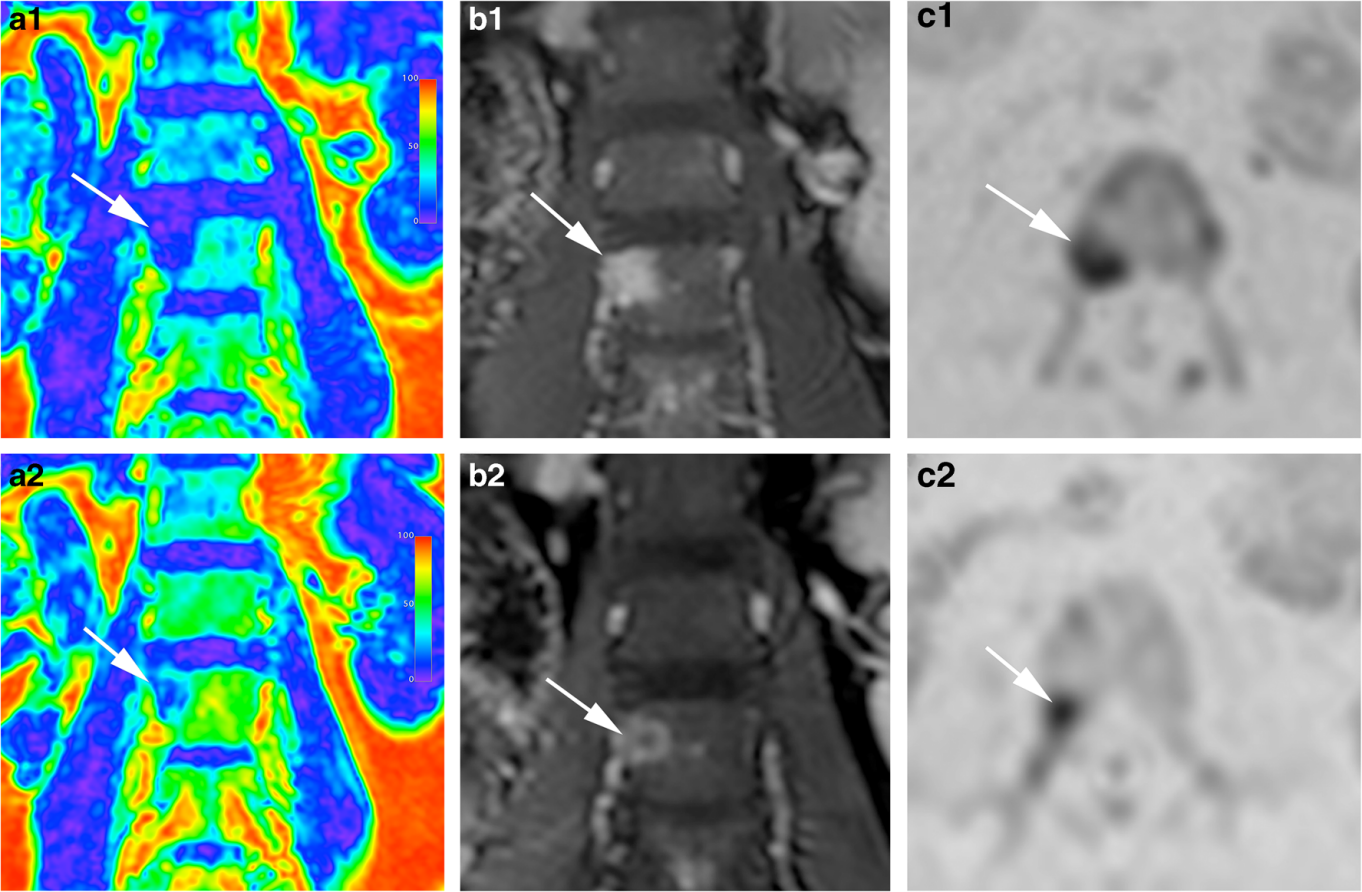
Representative images of the whole-body MRI scan of a 52-year-old female participant prior to (*a1*–*c1*) and following two cycles (*a2*–*c2*) of induction PAD (bortezomib, doxorubicin, dexamethasone) chemotherapy. *a1*, *a2* coronal signal fat fraction map; *b1*, *b2* coronal post-contrast water-only mDixon; *c1*, *c2* Axial *b*_1000_ diffusion-weighted MRI images depicting a focal lesion at the level of L3 vertebral body on the right side (*arrows*). Compared to baseline and following two cycles of treatment there were a 42.7% reduction in eTV, 51.7% reduction in ER, 25% increase in ADC and 80% increase in FF of the focal lesion

One (1/15) responder patient had a single FL and was therefore excluded from per patient analysis. An example of an FL at baseline and post-2nd cycle in a responding patient is presented in Fig. 3.

Following treatment, there was a significant increase in sFF for 13/14 responders (*p* < 0.01 to 0.03). For the remaining patient (1/14) with four FLs the change in sFF did not reach statistical significance (median sFF 0.27 and 0.36 a.u. at baseline and post-2nd cycle respectively, *p* = 0.12). There was no significant change in sFF following treatment in any of the six non-responder patients (*p* = 0.30 to 0.60).

ADC increased significantly following treatment in 7/14 responders (*p* < 0.01 to 0.04.). Six of 14 responders demonstrated no significant change of ADC (*p* = 0.12 to 1.0); and 1/14 demonstrated a significant decrease in ADC (median ADC of 1.08 and 0.68 × 10^−3^ mm^2^/s at baseline and post-2nd cycle, respectively, *p* = 0.01).

In the non-responder group, ADC did not change significantly following treatment in 4/6 patients (*p* = 0.50 to 0.97), and increased in 2/6 (*p* = 0.02 and 0.03).

The eTV decreased significantly in all six non-responders (*p* < 0.01 to 0.01) and in 13/14 responding patients (*p* < 0.01 to 0.02) with no significant change in the patient with four FLs (*p* = 0.62).

**Fig. 4:**
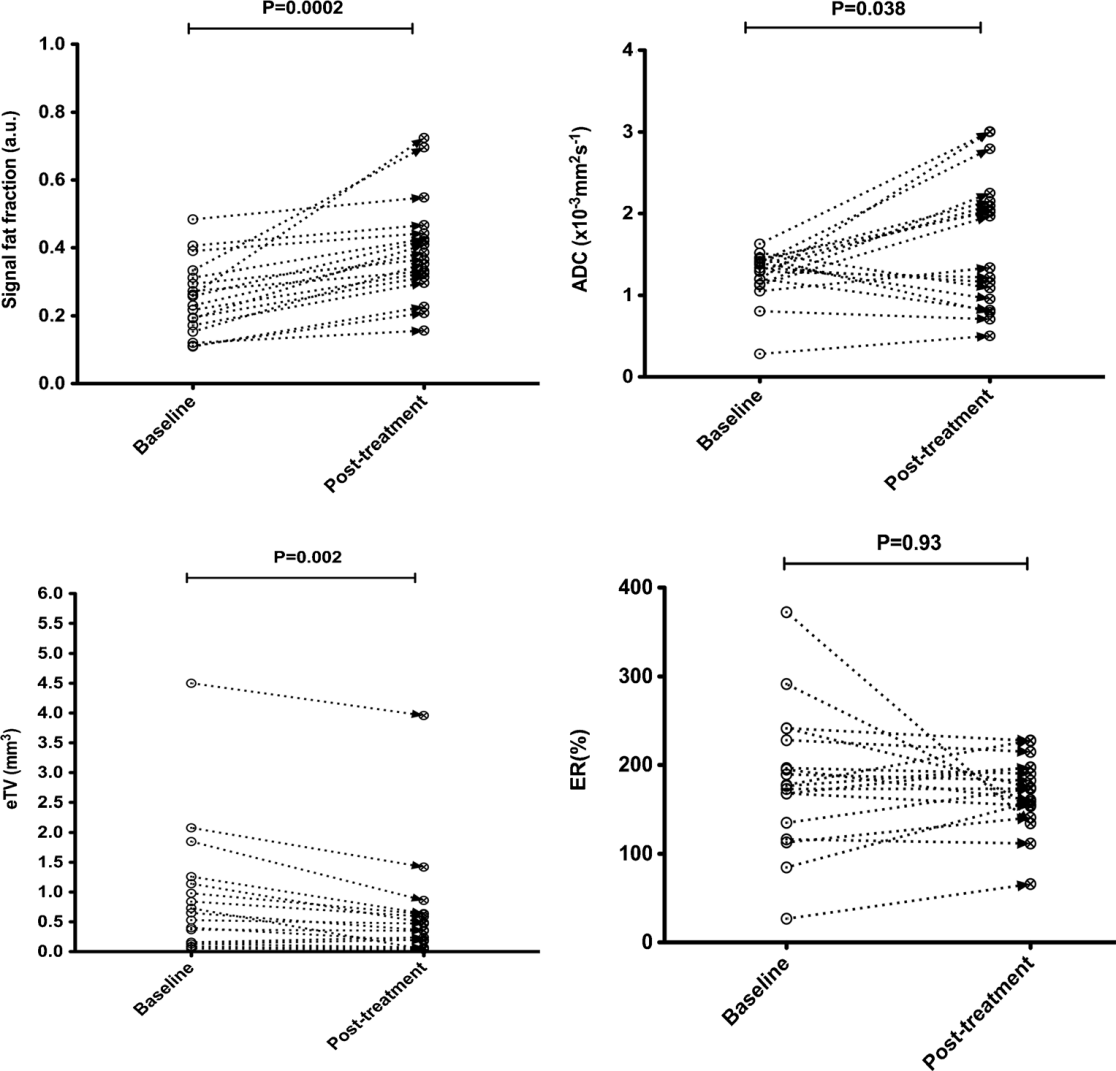
Per patient changes of signal fat fraction (sFF), apparent diffusion coefficient (ADC), estimated tumour volume (eTV) and enhancement ratio (ER) between baseline and post-2nd cycle scans. Subject is a 46-year-old male participant who achieved complete response (CR) after induction chemotherapy with PAD (bortezomib, doxorubicin, dexamethasone). Each *data point* is representative of a focal lesion at baseline and post-2nd cycle scan. Significant defined as *p* < 0.05

**Fig. 5:**
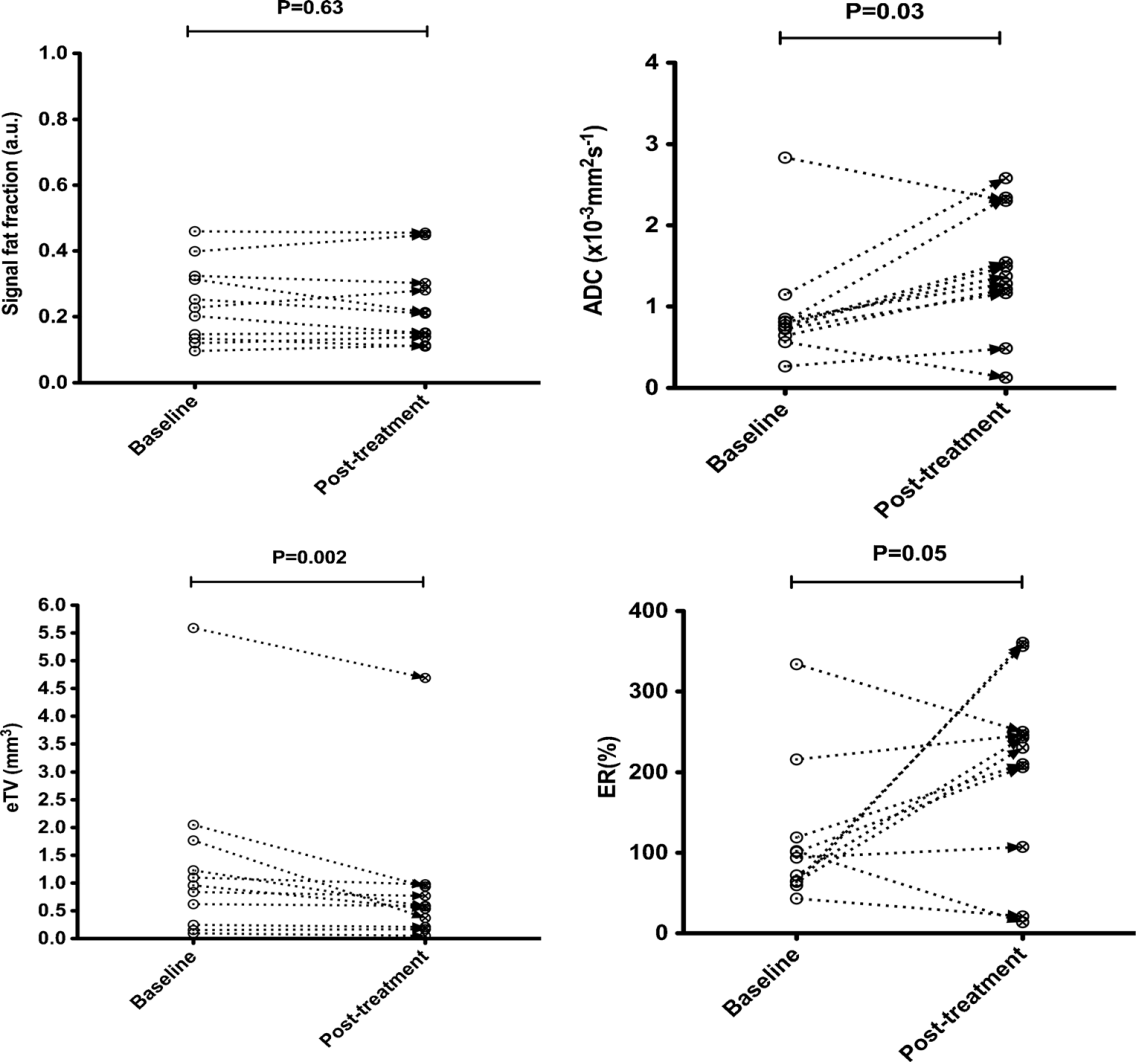
Per patient changes of signal fat fraction (sFF), apparent diffusion coefficient (ADC), estimated tumour volume (eTV) and enhancement ratio (ER) between baseline and post-2nd cycle scans. Subject is a 55-year-old male participant who achieved minimal response (MR) after induction chemotherapy with PAD (bortezomib, doxorubicin, dexamethasone). Each *data point* is representative of a focal lesion at baseline and post-2nd cycle scan. Significant defined as *p* < 0.05

In 6/14 responders, there was no significant change of ER following treatment (*p* = 0.20 to 0.93). The ER decreased significantly in 2/14 (*p* < 0.01) and significantly increased in 6/14 (*p* < 0.01 to 0.03). There was no significant change of ER in 3/6 (*p* = 0.14 to 0.31), and a significant increase of ER in 3/6 (*p* = 0.01 to 0.05) non-responder patients following treatment. Changes in sFF, ADC, eTV and ER for lesions in typical responder and non-responder patients are provided in Figs. 4 and 5, respectively.

Two patients (2/21) were excluded from analysis of the trochanteric sFF because of tumour involvement or artefact. There were no significant changes of sFF in normal-appearing greater trochanter (*n* = 19) following therapy (mean [standard deviation] of 0.97 [0.007] and 0.97 [0.007] at baseline and post-treatment, respectively, *p* = 0.16).

### ROC curves

The ROC curve analyses for eTV, ER, sFF and ADC using baseline, early post-treatment and percentage change following treatment as predictors of non-responding patients and AUC values are tabulated in Table 4. ROC analysis indicated that a threshold of 12.6% increase in sFF correctly identified response to treatment with 100% sensitivity and 100% specificity (AUC, 1.0).

**Table 4:** Patient cohort: univariate area under the curve (AUC) analysis of the imaging biomarkers at baseline, post-2nd cycle and their respective percentage changes

		AUC	Std.	Asymptomatic 95% CI	
				Lower bound	Upper bound
Baseline	eTV	0.55	0.15	0.26	0.84
	ER	0.48	0.13	0.23	0.72
	ADC	0.78	0.10	0.58	0.98
	sFF	0.78	0.11	0.56	1.00
Post-2nd cycle	eTV	0.56	0.15	0.26	0.86
	ER	0.51	0.13	0.26	0.76
	ADC	0.80	0.10	0.61	0.99
	sFF	0.73	0.12	0.49	0.97
Percentage changes	eTV	0.42	0.13	0.16	0.69
	ER	0.48	0.13	0.22	0.74
	ADC	0.70	0.13	0.44	0.96
	sFF	1.00	0.00	1.00	1.00

*eTV* estimated tumour volume, *ER* enhancement ratio, *ADC* apparent diffusion coefficient, *sFF* signal fat fraction, *AUC* area under the curve, *Std* standard deviation

### Biomarker repeatability (volunteer cohort)

**Fig. 6:**
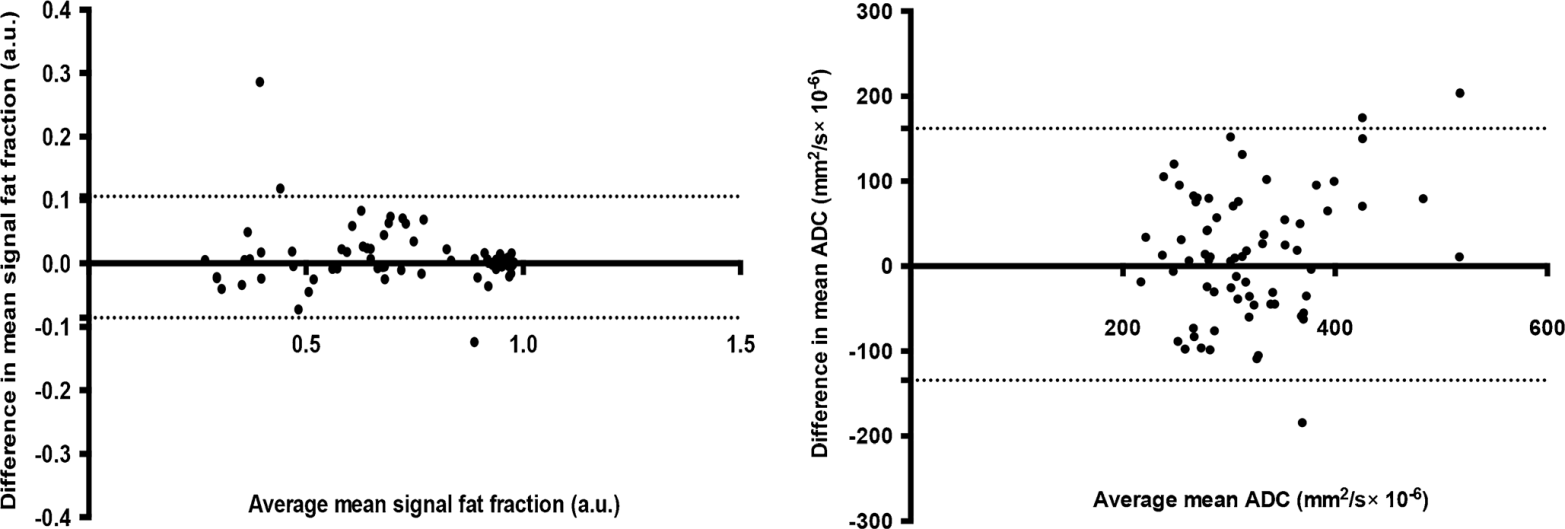
Bland–Altman plots of signal fat fraction (sFF) and apparent diffusion coefficient (ADC). For sFF, 95% limits of agreement were −0.085 to 0.105 (a.u.) with standard of bias of 0.048. For ADC, 95% limits of agreement were −134.3 to 162.1 (mm^2^/s × 10^−6^) with standard of bias of 75.61. There is a wider dispersion of values for ADC and mean differences between two measurements in each subject are closer to zero for sFF compared to ADC. The *dotted lines* represent 95% level of agreement

Median and interquartile ranges (IQR) of bone, splenic and subcutaneous adipose tissues ROIs for the first and second studies and ICC (95% confidence interval) between temporally separated studies are shown in Table 5. The ICC of sFF and ADC of normal bone were 0.98 (95% CI 0.96–0.98) and 0.47 (95% CI 0.26–0.63) respectively. The Bland–Altman plots for bone sFF and ADC measurements are presented in Fig. 6.

**Table 5:** Biomarker repeatability of normal volunteer cohort: median and interquartile range (IQR) of apparent diffusion coefficient and signal fat fraction of bone, apparent diffusion coefficient of spleen and signal fat fraction of subcutaneous adipose tissue for two scans and interclass correlation (ICC) and 95% confidence interval results are summarized

	Bone		Spleen	Adipose tissue
	ADC	sFF	ADC	sFF
1st scan	0.31 (0.28–0.36)	0.74 (0.55–0.93)	0.75 (0.74–0.79)	0.95 (0.93–0.96)
2nd scan	0.31 (0.26–0.35)	0.70 (0.56–0.94)	0.77 (0.75–0.80)	0.94 (0.93–0.96)
ICC (95% CI)	0.47 (0.26–0.63)	0.98 (0.96–0.98)	0.83 (0.47–0.95)	0.93 (0.75–0.98)

*ADC* apparent diffusion coefficient, *sFF* signal fat fraction, *ICC* interclass correlation, *CI* confidence interval

## Discussion

WB-MRI is increasingly used for initial evaluation of patients with MM. Recently, qualitative and quantitative assessments of WB-MRI have yielded promising results for monitoring response in MM [9, 13, 14].

Our study demonstrates that MR-derived sFF is a reliable and quantifiable technique and that its changes following two chemotherapy cycles were a consistent and accurate classifier of treatment response.

Our findings on WB-MRI confirm previous reports on repeatability and reproducibility of sFF of lumbar spine [22] and subcutaneous adipose tissue [23].

We observed a significant increase in sFF in responders (13/14) compared to no significant change in non-responders (6/6).

Few studies have investigated sFF quantification of bone marrow in MM. Takasu et al. [24] demonstrated a significant decrease in lumbar spine fat fraction of 24 MM patients, compared to healthy volunteers, monoclonal gammopathy of undetermined significance and asymptomatic MM patients, using iterative decomposition of water and fat with echo asymmetric and least-squares estimation (IDEAL) [25] MRI. In their cohort, discriminant analysis of sFF showed that 92% of patients were classified correctly into symptomatic or non-symptomatic MM groups.

FLs’ response sometimes does not reflect as a change in lesion diameter, but rather progressive fading of the marrow abnormalities and return to normal marrow signal intensity within the lesion [26] as reflected in our results.

Although we found a significant increase of ADC in our responder group we observed more variation in per patient analysis than reported previously on 1.5-T DW MR imaging [13, 15]. The temporal changes of ADC following myelomatous infiltration and treatment are complex and affected by several factors such as the amount of fatty (yellow) marrow, cell size, bulk flow in capillaries and cellular architecture [27]. Furthermore, the direction and magnitude of ADC changes may depend on the stage of bone remodelling cycle captured in individual patients, and individual lesions.

We observed a significant decrease in eTV following treatment in almost all the patients (19/20) but found no significant change in FL enhancement following treatment, concurring with a previous report [14].

Persistent increased angiogenesis previously reported in MM following successful treatment might explain observed ER changes in our cohort [28]. Furthermore, perceived changes in eTV could highlight inadequacy of single axis size measurement in FLs as a measure of response/progression.

A prerequisite of a successful response biomarker, in addition to changing with treatment, is clinically acceptable repeatability. We determined the repeatability of bone ADC and sFF measurements in normal volunteers, and showed that sFF has excellent repeatability (ICC of 0.98) compared with moderate repeatability of ADC (ICC bone 0.47). A separate splenic ROI was used as DWI is normally performed with fat-suppression which may reduce the signal-to-noise ratio on DWI images in normal marrow and affect ADC calculation and thus repeatability. In the spleen (where DWI signal is high) ADC has good repeatability.

The repeatability of sFF measurements, its modifiability with treatment together with the significant increase with successful treatment, strongly supports the use of sFF as a biomarker of treatment response in patients with MM.

Our study has some limitations. Although we observed differential responses between lesions within individual patients, we could not confirm response on a per lesion basis, as our reference standard was defined by a global patient-based response. It is possible that individual lesions that do not appear to be responding, in accordance with our observed MRI biomarker change associated with global response, are the sites of resistance to therapy. Further temporal follow-up with and without individual lesion biopsy is necessary to explore this hypothesis.

Because the focal pattern constitutes the most common pattern of bone marrow involvement in MM [29], as reflected in our cohort, our findings may not be generalizable to patients with diffuse-only pattern of disease.

Additionally, the second time point (post-2nd cycle) was an arbitrary choice in our study as there is currently no consensus on the optimal point for early/late follow-up imaging in MM using WB-MRI.

We acknowledge that changes as early as 1-month post chemotherapy have been documented for ADC in MM patients [21] however, the most optimal post-treatment interval with greatest sensitivity to separate responders and non-responders patients is yet to be confirmed.

As we required whole-body coverage, we were limited to static CE MRI following a constant administration of 20 ml of gadolinium as opposed to dynamic CE MR imaging. In static CE MRI, the final gadolinium concentration at equilibrium state is measured, which provides less discriminative information than dynamic CE (DCE) imaging, as previously reported when applied to the spine for prognostic categorization of MM patients [30].

Finally, we used our volunteer cohort to assess quantitative parameter repeatability, and we acknowledge that our volunteer cohort was not sex- and age-matched to the patient cohort.

Our observations require subsequent confirmation in a larger patient cohort. Imaging was performed on a single MRI scanner and so biomarker generalizability between platforms and institutions/settings is untested. However, the pulse sequences used are routinely available on all commercially available scanners, quantification is relatively simple, and repeatability of sFF (our best response biomarker) is almost perfect.

We envisage that a 10-min WB-MRI mDixon imaging could provide a safe, fast and cost-effective technique with simple and reliable quantification of sFF to assess treatment response accurately, and has the potential to aid treatment stratification by early identification of poorly responding patients. Moreover, sFF imaging may demonstrate heterogeneity of lesion response, which, if verified, may identify lesions containing resistant disease, facilitating targeted biopsy and guiding further therapy. Evolving risk stratification methods are integrating more advanced and sophisticated tests (e.g. cytogenetic analysis) for assessment of MM and WB-MRI sFF could potentially provide added value in making clinical management decisions within this heterogeneous disease.

In conclusion, we found that WB-MRI sFF assessment using a simple MRI technique provides a reliable MRI biomarker for assessing therapeutic response and potentially discriminates clinical outcome in patients with symptomatic multiple myeloma.

## References

[1] Dinter DJ, Neff WK, Klaus J (2009). Comparison of whole-body MR imaging and conventional X-ray examination in patients with multiple myeloma and implications for therapy. Ann Hematol.

[2] Baur-Melnyk A, Buhmann S, Becker C (2008). Whole-body MRI versus whole-body MDCT for staging of multiple myeloma. Am J Roentgenol.

[3] Shortt CP, Gleeson TG, Breen KA (2009). Whole-body MRI versus PET in assessment of multiple myeloma disease activity. Am J Roentgenol.

[4] Bäuerle T, Hillengass J, Fechtner K (2009). Multiple myeloma and monoclonal gammopathy of undetermined significance: importance of whole-body versus spinal MR imaging. Radiology.

[5] Dimopoulos MA, Hillengass J, Usmani S (2015). Role of magnetic resonance imaging in the management of patients with multiple myeloma: a consensus statement. J Clin Oncol.

[6] National Institute for Health and Care Excellence (NICE) (2016). NICE guideline, recommendation on imaging investigation. https://www.nice.org.uk/guidance/ng35/chapter/recommendations#imaging-investigations. Accessed 22 Feb 2016.

[7] Durie BG, Harousseau JL, Miguel JS (2006). International uniform response criteria for multiple myeloma. Leukemia.

[8] Hillengass J, Fechtner K, Weber MA (2010). Prognostic significance of focal lesions in whole-body magnetic resonance imaging in patients with asymptomatic multiple myeloma. J Clin Oncol.

[9] Hillengass J, Ayyaz S, Kilk K (2012). Changes in magnetic resonance imaging before and after autologous stem cell transplantation correlate with response and survival in multiple myeloma. Haematologica.

[10] Moulopoulos LA, Dimopoulos MA, Alexanian R (1994). Multiple myeloma: MR patterns of response to treatment. Radiology.

[11] O’Connor JPB, Jackson A, Asselin MC (2008). Quantitative imaging biomarkers in the clinical development of targeted therapeutics: current and future perspectives. Lancet Oncol.

[12] Smith JJ, Sorensen AG, Thrall JH (2003). Biomarkers in imaging: realizing radiology future. Radiology.

[13] Horger M, Weisel K, Horger W (2011). Whole-body diffusion-weighted MRI with apparent diffusion coefficient mapping for early response monitoring in multiple myeloma: preliminary results. Am J Roentgenol.

[14] Lin C, Luciani A, Belhadj K (2010). Multiple myeloma treatment response assessment with whole-body dynamic contrast-enhanced MR imaging. Radiology.

[15] Giles SL, Messiou C, Collins DJ (2014). Whole-body diffusion-weighted MR imaging for assessment of treatment response in myeloma. Radiology.

[16] Dutoit JC, Vanderkerken MA, Verstraete KL (2013). Value of whole body MRI and dynamic contrast enhanced MRI in the diagnosis, follow-up and evaluation of disease activity and extent in multiple myeloma. Eur J Radiol.

[17] Dutoit JC, Claus E, Offner F (2016). Combined evaluation of conventional MRI, dynamic contrast-enhanced MRI and diffusion weighted imaging for response evaluation of patients with multiple myeloma. Eur J Radiol.

[18] Angtuaco EJ, Fassas AB, Walker R (2004). Multiple myeloma: clinical review and diagnostic imaging. Radiology.

[19] Durie BG, Kyle RA, Belch A (2003). Myeloma management guidelines: a consensus report from the Scientific Advisors of the International Myeloma Foundation. Hematol J.

[20] Punwani S, Taylor SA, Saad ZZ (2013). Diffusion-weighted MRI of lymphoma: prognostic utility and implications for PET/MRI?. Eur J Nucl Med Mol Imaging.

[21] Messiou C, Giles S, Collins DJ (2012). Assessing response of myeloma bone disease with diffusion-weighted MRI. Br J Radiol.

[22] Maas M, Akkerman EM, Venema HW (2001). Dixon quantitative chemical shift MRI for bone marrow evaluation in the lumbar spine: a reproducibility study in healthy volunteers. J Comput Assist Tomogr.

[23] Berglund J, Johansson L, Ahlstrom H (2010). Three-point Dixon method enables whole-body water and fat imaging of obese subjects. Magn Reson Med.

[24] Takasu M, Tani C, Sakoda Y (2012). Iterative decomposition of water and fat with echo asymmetry and least-squares estimation (IDEAL) imaging of multiple myeloma: Initial clinical efficiency results. Eur Radiol.

[25] Reeder SB, Pineda AR, Wen Z (2005). Iterative decomposition of water and fat with echo asymmetry and least-squares estimation (IDEAL): application with fast spin-echo imaging. Magn Reson Med.

[26] Dutoit JC, Verstraete KL (2016). MRI in multiple myeloma: a pictorial review of diagnostic and post-treatment findings. Insight Imaging.

[27] Messiou C, Kaiser M (2015). Whole body diffusion weighted MRI–a new view of myeloma. Br J Haematol.

[28] Rajkumar SV, Fonseca R, Witzig TE (1999). Bone marrow angiogenesis in patients achieving complete response after stem cell transplantation for multiple myeloma. Leukemia.

[29] Moulopoulos LA, Gika D, Anagnostopoulos A (2005). Prognostic significance of magnetic resonance imaging of bone marrow in previously untreated patients with multiple myeloma. Ann Oncol.

[30] Merz M, Moehler TM, Ritsch J (2016). Prognostic significance of increased bone marrow microcirculation in newly diagnosed multiple myeloma: results of a prospective DCE-MRI study. Eur Radiol.

